# Plasma Nanocoatings Developed to Control the Shear Strength of Polymer Composites

**DOI:** 10.3390/polym11071188

**Published:** 2019-07-15

**Authors:** Milan Zvonek, Veronika Sirjovova, Martin Branecky, Tomas Plichta, Josef Skacel, Vladimir Cech

**Affiliations:** 1 Institute of Materials Chemistry, Faculty of Chemistry, Brno University of Technology, Purkynova 118, 61200 Brno, Czech Republic; 2 Department of Microelectronics, Faculty of Electrical Engineering and Computer Science, Brno University of Technology, Technicka 3058/10, 616 00 Brno, Czech Republic

**Keywords:** plasma nanocoatings, glass fibers, polymer-matrix composites, interface/interphase, shear strength, adhesion

## Abstract

All reinforcements for polymer-matrix composites must be coated with a suitable material in the form of a thin film to improve compatibility and interfacial adhesion between the reinforcement and the polymer matrix. In this study, plasma nanotechnology was used to synthetize such functional nanocoatings using pure tetravinylsilane (TVS) and its mixtures with oxygen gas (O_2_) as precursors. The plasma-coated glass fibers (GFs) were unidirectionally embedded in a polyester resin to produce short composite beams that were analyzed by a short-beam-shear test to determine the shear strength characterizing the functionality of the nanocoatings in a GF/polyester composite. The developed plasma nanocoatings allowed controlling the shear strength between 26.2–44.1 MPa depending on deposition conditions, i.e., the radiofrequency (RF) power and the oxygen fraction in the TVS/O_2_ mixture. This range of shear strength appears to be sufficiently broad to be used in the design of composites.

## 1. Introduction

Atomically accurate synthesis of new materials and devices with complex application-tailored structures is essential for the development of sophisticated nanotechnology-based products [1]. Nowadays, wet chemical technologies for the production of this type of material lack the precision to determine their properties and the synthesized materials contain numerous imperfections at the atomic level. Using bottom-up approaches, which use small molecule fragments or individual atoms as building blocks (plasma nanotechnology) is an attractive approach to synthesizing very complex and yet well-defined material structures. The synthesis of functional nanocoatings with controlled mechanical and chemical properties is an example of highly sophisticated materials. Such tailored nanocoatings are required for controlled interphase in hybrid materials, such as polymer-matrix composites and nanocomposites reinforced by micro- or nano-fibers and micro- or nano-particles. Plasma nanotechnology using atomic processes seems to be the right tool for the synthesis of such nanocoatings.

All reinforcing fibers and particles must be coated as part of the manufacturing process to improve their chemical and physical compatibility and control adhesion between the composite constituents. This type of coating is usually referred to as sizing when using wet chemical processes. The interphase region [2] between the surface of reinforcement and the polymer matrix is responsible for the stress transfer from the flexible matrix to the rigid reinforcement and for controlling the micromechanics of failure and thus durability of the composite material [3,4,5]. The surface modification of fibers [6,7,8,9,10,11] and particles [12,13,14] is a highly topical issue in the development of polymer-matrix composites determined for advanced applications.

Plasma coating is an alternative technology to wet chemical processes, which can significantly improve the functionality of reinforcements in polymer-matrix composites [15,16,17]. Plasma nanotechnology operated as plasma-enhanced chemical vapor deposition (PECVD) [18] employs non-thermal plasma, where the temperature of ions and neutral particles is less than 400 K, which retains the bulk properties even of polymer reinforcements. Plasma nanocoatings are synthetized from fragments of precursor molecules, resulting in a chemical structure that is responsible for the mechanical properties of coatings depending on deposition conditions [19,20]. This technology allows the synthesis of materials with chemical and mechanical properties adapted to the controlled interphase for a specific composite system determined by the reinforcement and the polymer matrix.

In this study, we focused on the plasma coating of continuous glass fibers used as reinforcements for polyester resin (polymer matrix). The idea was to develop compatible plasma nanocoatings with controlled mechanical properties that are covalently bonded to the surface of glass fiber (GF) and are covalently bonded to the polyester resin during the curing process. Based on previous studies, the tetravinylsilane (TVS) precursor was used for the synthesis of plasma nanocoatings because of the vinyl group responsible for covalent bonding to the polyester resin. The TVS precursor was also mixed with gaseous oxygen to oxidize plasma nanocoatings and create new chemical species to control the interphase in GF/polyester composite. The shear strength of polymer composites with plasma-coated GFs was investigated depending on the deposition conditions and the mechanical/chemical properties of the deposited nanocoatings.

## 2. Materials and Methods

### 2.1. Plasma Nanotechnology

The novel plasma device with a 1 m tubular PECVD reactor was developed and tested to determine the range of operation conditions for plasma pretreatment and plasma coating of continuous fibers. All technical details of the plasma device using radiofrequency (RF, 13.56 MHz) glow discharge are given in Ref. [21]. The cylindrical shape of the plasma reactor with axially symmetrical plasma is important for uniform plasma density around the fiber or bundle of fibers positioned along the axis. This plasma apparatus is used to develop suitable plasma nanocoatings that subsequently could be employed for the continuous surface modification of fibers in a roll-to-roll device, designed on an industrial scale, with more plasma reactors performing specific operations (plasma pretreatment, plasma coating, post-treatment of fibers), or several reactors can use the same operation to increase the fiber winding speed [21].

Unsized continuous GFs with 1,600 filaments in a bundle (1200 tex) were plasma coated in this study. The unsized and sized fibers of E-glass with a mean diameter of 19 µm were provided by Saint-Gobain Adfors CZ s.r.o. (Litomysl, Czech Republic). A 6 m bundle of fibers was placed along the tube axis, using a special glass frame. Selected analyzes of plasma nanocoatings were made for coatings deposited on flat substrates that were more suitable for thin film characterization. Therefore, soda-lime float glass plates with dimensions of 1.0 × 26 × 76 mm^3^ from Knittel Glaeser (Braunschweig, Germany) and double-side polished silicon wafers (100) of 0.6 × 10 × 10 mm^3^ covered with a native SiO_2_ layer from ON Semiconductor (Roznov pod Radhostem, Czech Republic) were used as flat substrates. Flat substrates on special glass stands can be positioned at any position along the axis of the cylindrical plasma reactor.

After loading the substrate into the plasma reactor, the reactor was closed and pumped to a basic pressure of 5 × 10^−4^ Pa. Then, the deposition system was flushed with argon gas (99.999% purity, Linde Gas (Brno, Czech Republic)) using a flow rate of 10.0 sccm at a pressure of 5.7 Pa for 5 min. Any substrate (GF, glass plate, silicon wafer) was pretreated with oxygen plasma (10.0 sccm, 5.0 Pa) at an RF power of 30 W (flat substrate) or 100 W (GF) for 10 or 30min, respectively. Oxygen gas (99.99% purity) was supplied by Linde Gas (Brno, Czech Republic). Oxygen plasma pretreatment is important for cleaning the substrate surface from adsorbed gases and activating it to improve plasma coating adhesion. Then, a pure TVS precursor or TVS mixed with oxygen gas (0–0.71 oxygen fraction) was used to deposit the plasma nanocoatings under a continuous wave (RF power: 1.0–200 W) or pulsed plasma (effective power: 2.0–150 W). TVS precursor, Si–(CH=CH_2_)_4_, of 97% purity was supplied by Sigma Aldrich (Prague, Czech Republic). After plasma coating, the deposition system was again flushed with argon gas (10.0 sccm, 5.7 Pa) for 60 min and then evacuated to basic pressure. After 12 h under vacuum, the plasma reactor was flooded by air to atmospheric pressure and the coated substrate was removed to be characterized or embedded in the polymer composite.

### 2.2. Plasma Nanocoatings Characterization

Plasma nanocoatings deposited on flat substrates (glass plate, silicon wafer) were used to characterize the coating thickness. Five scratches in the coating to the substrate were used to evaluate the mean coating thickness using mechanical profilometry (DektakXT stylus profiler (Bruker, Billerica, MA, USA)) with a 25 μm diameter stylus and a low normal force corresponding to 2 mg. The mean deposition rate for plasma coating was estimated as the ratio between the coating thickness and deposition time.

Adhesion of plasma nanocoatings to glass plates were characterized by nanoscratch test using a 2D TriboScope (Hysitron, Minneapolis, MN, USA) attached to an NTegra Prima Scanning Probe Microscope (NT-MDT, Russia). Nanoscratches in plasma coating with a thickness of 0.10 µm were made with a conical diamond indenter with a tip radius of 1.0 µm under normal load ranging from 1 μN to 6 mN. Ten 10 µm scratches achieved in 30 s were used to record normal and lateral forces. A detailed analysis of nanoscratch measurements for coatings on single GFs and planar glass was previously published [22].

Chemical analysis of 0.10 µm-thick plasma coatings on infrared-transparent silicon wafers was performed under vacuum (160 Pa) using a VERTEX 80v, vacuum Fourier transform infrared (FTIR) spectrometer (Bruker Optics, USA). The spectrometer was equipped with an internal air cooled mid-IR source with F350 MIR polarizer, standard MIR KBr beamsplitter (T304/8), and DLaTGS D301 detector. Transmission spectra were recorded at a wavenumber of 4000–500 cm^−1^ using 256 scans and 4 cm^−1^ spectral resolution. The infrared (IR) spectrum of the plasma nanocoating was obtained by subtracting the spectrum of the bare silicon wafer from the recorded spectrum.

A phase-modulated spectroscopic ellipsometer UVISEL (Jobin-Yvon, France) was used to characterize the optical properties of plasma nanocoatings deposited on silicon wafers. Measurements were carried out at an incidence angle of 70° and 100 × 300 μm^2^ spot size using a wavelength of 250–830 nm with a step of 5 nm. The dispersion dependence of the dielectric function was fitted using the five-parametric Tauc-Lorentz formula suitable for amorphous dielectrics. Details of the ellipsometric spectrum analysis can be found in the previous publication [20].

### 2.3. Composite Short Beams and Their Shear Strength

The GF bundles were manually inserted into the mold, impregnated with unsaturated polyester resin (Viapal HP 349 F, Sirca S. p. A., Italy), and cured in an oven first at 100 °C for 30 min and then 140 °C for 1 h. The fiber volume fraction was 35%. The cured composite was then cut into short beams of 18 × 10 × 3 mm^3^. The short-beam-shear (SBS) test (ASTM D 2344 [23]) was used to determine the short-beam strength, *F*^sbs^, given by [23]

(1) $$F^{sbs}=0.75\times \frac{P_{m}}{b\times h},$$

where *P*_m_ corresponds to the maximum load measured by a universal test machine Z010/TH2A (Zwick, Germany) at a crosshead speed of 1.0 mm min^−1^, *b* and *h* corresponds to the width and thickness of the short beam, respectively. Eight composite beams were used to determine the mean value and its standard deviation. The composite short beams after fracture were sputtered with gold to improve their surface conductivity, and then observed using a scanning electron microscope (SEM) (Philips XL 30/EDAX/Microspec).

## 3. Results and Discussion

### 3.1. Selection of Deposition Conditions

**Figure 1:**
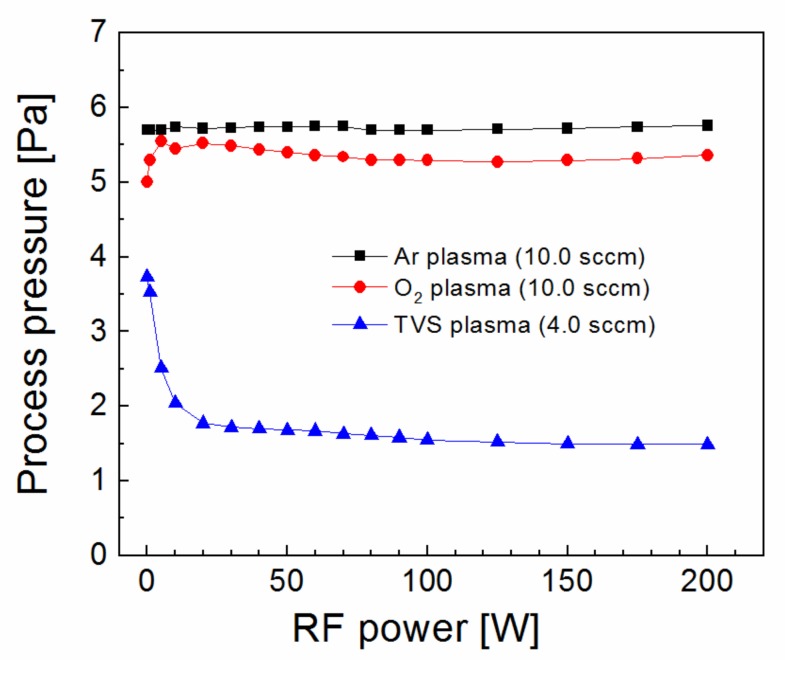
Process pressure as a function of radiofrequency (RF) power for argon, oxygen, and tetravinylsilane (TVS) plasma at a given flow rate.

The process pressure as a function of RF power was characterized for argon (10.0 sccm), oxygen (10.0 sccm), and TVS (4.0 sccm) plasma in Figure 1. The basic process pressure, when the plasma was turned off, was set to 5.7 Pa using a throttle valve [21] and the resulting process pressure was independent of RF power during the argon discharge, as expected. While, the process pressure for oxygen plasma increased slightly to 5.5 Pa (5 W) relative to a basic process pressure of 5.0 Pa, due to dissociation of oxygen molecules to reactive oxygen atoms. This oxygen plasma at an RF power of 30 or 100 W was used for substrate pretreatment. The basic process pressure for the TVS precursor at 3.8 Pa was significantly reduced to 1.5 Pa, which is characteristic of the precursor-deficient mode [24]. This means that the TVS molecules were dissociated into smaller radicals that were highly reactive and resulted in their recombination on the surface of the growing coating. The largest decrease in the process pressure can be observed between 0 and 20 W.

**Figure 2:**
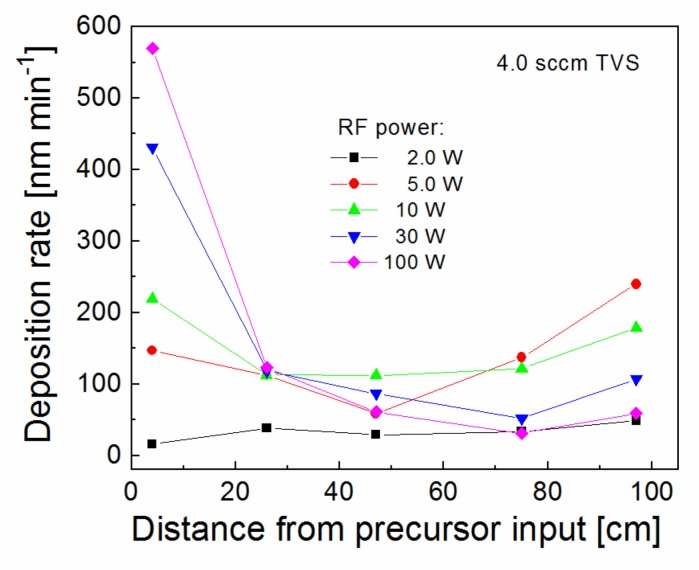
Deposition rate along the tubular reactor for plasma nanocoatings deposited at a TVS flow rate of 4.0 sccm and different RF powers.

**Figure 3:**
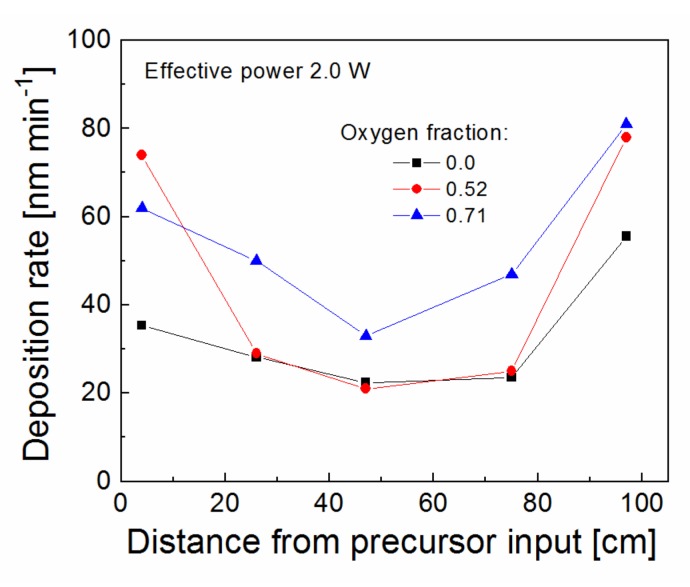
Deposition rate along the tubular reactor for plasma nanocoatings deposited from the TVS/O_2_ mixture at an effective power of 2.0 W and different oxygen fractions.

**Figure 4:**
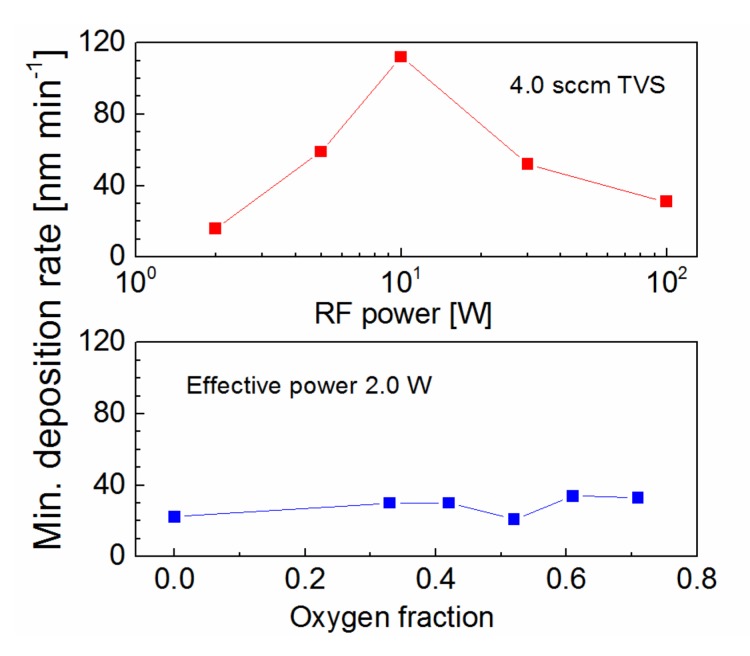
Minimum deposition rate dependent on RF power for plasma nanocoatings deposited from pure TVS at a flow rate of 4.0 sccm (upper part) and dependent on oxygen fraction for nanocoatings deposited from the TVS/O_2_ mixture at an effective power of 2.0 W (bottom part).

The deposition rate along the axis of tubular reactor was evaluated in Figure 2 for a TVS flow rate of 4.0 sccm, but a different RF power of 2.0–100 W. The TVS vapors enter the reactor from one end of the tube and exit at the other end connected to the pumping system [21]. It can be seen in Figure 2 that the deposition rate is not constant along the tubular reactor, especially in the case of higher power. A deposition rate of 570 nm min^−1^ at the precursor input dropped to a minimum of 31 nm min^−1^ at a distance of 75 cm from the precursor input for 100 W. In the case of 2.0 W, the deposition rate only slightly varied between 16–49 nm min^−1^ along the tube. It has been found that plasma nanocoatings deposited from a mixture of a TVS precursor with oxygen gas at low power efficiently affect the interfacial properties of polymer composites [25], since the low power resulted in reduced network crosslinking, supported by increased oxygen fraction, which caused a decline of nanocoating mechanical properties favorable to reduce the shear stress across the composite interphase [16]. For this reason, pulsed plasma at 2.0 W and various oxygen fractions (0–0.71) in TVS/O2 mixture were tested for thin film deposition (Figure 3). The deposition rate along the tubular reactor varied from 21 to 81 nm min^−1^ for an oxygen fraction between 0 and 0.71with a minimum close to half of the reactor. The minimum deposition rate is important to ensure full coverage of all fibers in the bundle by plasma coating to effectively control the interphase in the polymer composite. This parameter was characterized depending on the RF power for pure TVS precursor and depending on the oxygen fraction for an effective power of 2.0 W in Figure 4. The minimum deposition rate ranged broadly from 16 to 112 nm min^−1^, depending on the RF at the top of Figure 4, but was similar (21–34 nm min^−1^) for different oxygen fractions in the lower part of Figure 4. For TVS/O2 mixture, the TVS flow rate was constant at 4.0 sccm and the oxygen flow rate increased as follows: 0 sccm (zero oxygen fraction), 2.0 sccm (0.33), 2.9 sccm (0.42), 4.3 sccm (0.52), 6.2 sccm (0.61), and 10.0 sccm (0.71).

### 3.2. Mechanical, Optical, and Chemical Properties of Plasma Nanocoatings

**Figure 5:**
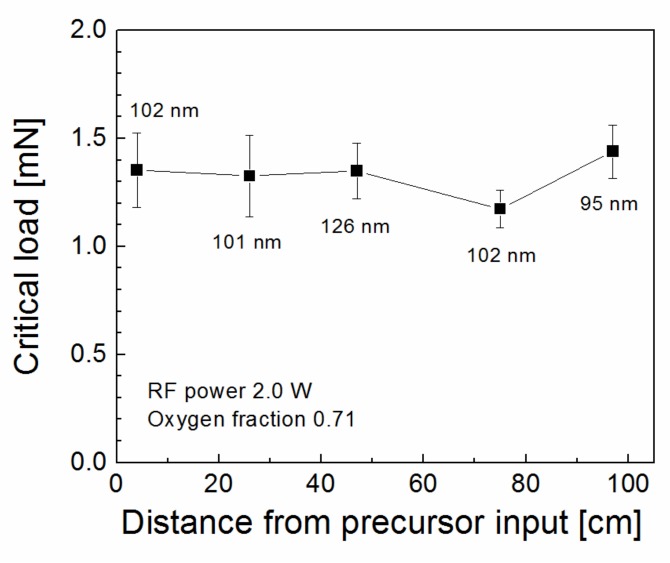
Critical load corresponding to plasma nanocoatings on silicon wafers distributed along the tubular reactor for selected deposition conditions. Nanocoating thickness is given.

**Figure 6:**
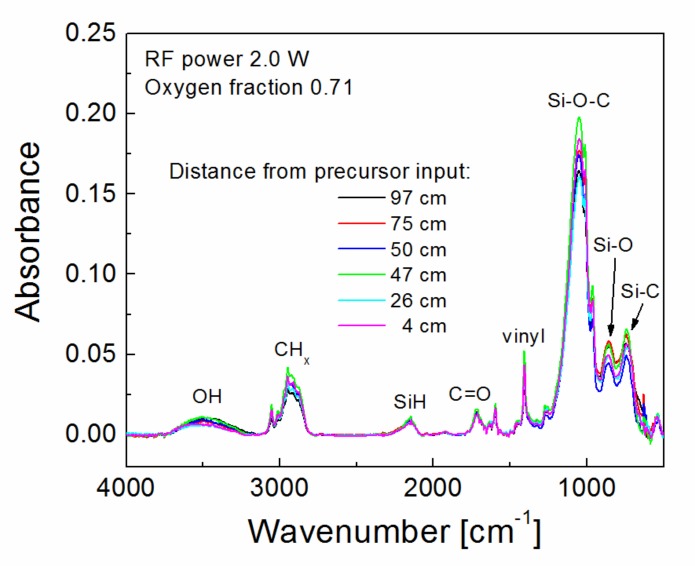
Infrared spectra for plasma nanocoatings on silicon wafers distributed along the tubular reactor for selected deposition conditions.

A strong correlation was demonstrated between the shear strength of the polymer composite reinforced with plasma-coated GFs and the nanocoating adhesion [25], because the shear failure of the composite was shown to be controlled by interfacial adhesion at the nanocoating/glass interface. Importantly, the adhesion of the nanocoating measured on GF is consistent with the adhesion measured on the glass plate using the nanoscratch test [22]. The critical load required to remove the nanocoating from the glass surface is used to characterize its level of adhesion. This means that the adhesion of the nanocoating increases when the critical load increases. Adhesion of plasma nanocoatings deposited on silicon wafers at selected deposition conditions (2.0 W, 0.71 oxygen fraction) was characterized by a nanoscratch test for samples distributed along the tubular reactor (Figure 5). The silicon wafer covered with the native silicon dioxide layer is fully comparable to the glass plate in terms of nanocoating adhesion [25]. The critical load depends on the nanocoating thickness [26,27] and, therefore, the deposited nanocoatings had a similar thickness of about 0.10 µm as shown in Figure 5. The critical load fluctuated only slightly 1.2–1.4 mN along the tubular reactor (Figure 5), indicating the same adhesion of nanocoatings independent of the deposition rate, see the blue triangles in Figure 3. FTIR spectra of the same samples are given in Figure 6 to compare the chemical structure of the deposited nanocoatings along the tubular reactor. Assignment of absorption bands to chemical species [28,29] is indicated directly in the spectra. It can be seen that the FTIR spectra are very similar, demonstrating the same chemical character of the deposited nanocoatings, regardless of position along the tube, and thus independent of the deposition rate similar as nanocoating adhesion. The polar groups, hydroxyl (–OH) and carbonyl (–C=O), improve wettability of the nanocoatings with polyester resin and the vinyl groups on the surface of the nanocoatings are responsible for covalent bonding with the polyester resin during the curing process [30]. It is known that a high concentration of Si–O–C species is responsible for the adhesion of the nanocoating to the glass surface [16,17].

**Figure 7:**
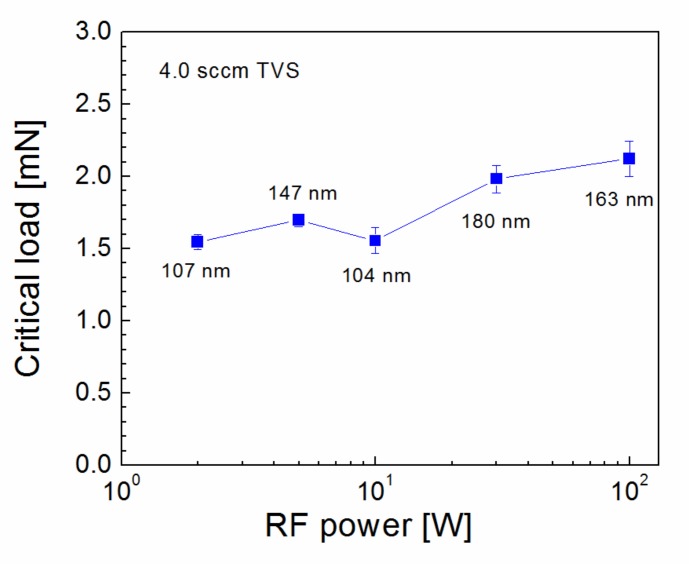
Critical load as a function of RF power for plasma nanocoatings deposited on silicon wafers from pure TVS precursor. Nanocoating thickness is given.

**Figure 8:**
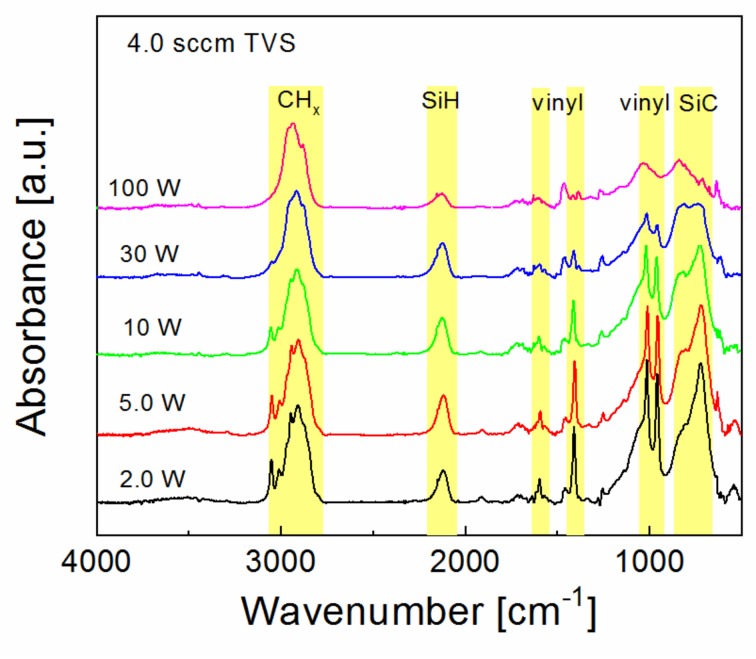
Infrared spectra for plasma nanocoatings deposited at different RF powers from pure TVS precursor.

Nanocoating adhesion as a function of RF power for plasma nanocoatings deposited on silicon wafers from pure TVS precursor is characterized in Figure 7. The critical load increased slightly from 1.5 to 2.1 mN with enhanced RF power (2.0–100 W), but higher critical load values may be affected by thicker coating. FTIR spectra corresponding to the samples of Figure 7 are shown in Figure 8. It is evident that the concentration of the vinyl groups (1590, 1402, 1007, and 951 cm^−1^) decreased considerably with enhanced RF power and no hydroxyl and carbonyl groups were present in the plasma nanocoatings, which could mean reduced interfacial adhesion at the polyester/nanocoating interface, when such a nanocoating is used for surface modification of GFs in GF/polyester composite. The absence of Si–O–C species in nanocoatings should also result in lower adhesion at the nanocoating/glass interface. However, we can expect the formation of the Si–O–C species only at this interface due to the hydroxyl groups typically present on any silicon dioxide surface. This could be a reason for good adhesion even for plasma nanocoatings deposited from a pure TVS precursor. According to Ref. [15], the reduced concentration of silicon atoms from 9 to 5 at.% and hydrogen atoms from 55 to 53 at.% at the expense of increased carbon concentration from 36 to 42 at.% with enhanced RF power should be responsible for the decreasing intensity of absorption band assigned to the Si-H group in Figure 8.

**Figure 9:**
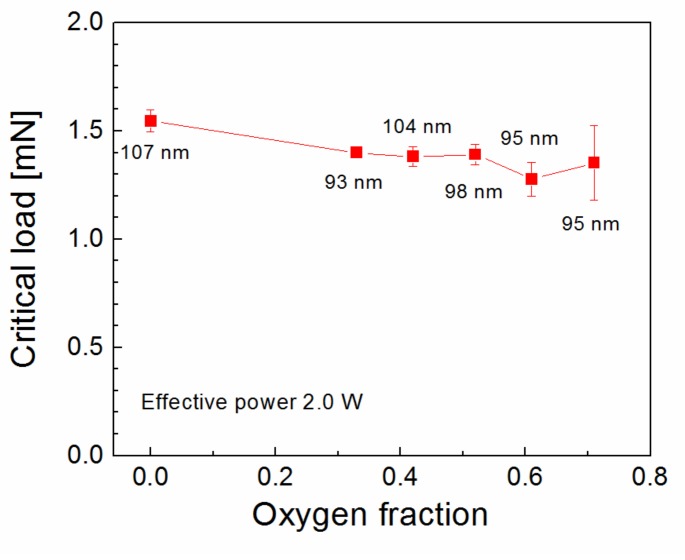
Critical load as a function of oxygen fraction for plasma nanocoatings deposited on silicon wafers from TVS/O_2_ mixture. Nanocoating thickness is given.

**Figure 10:**
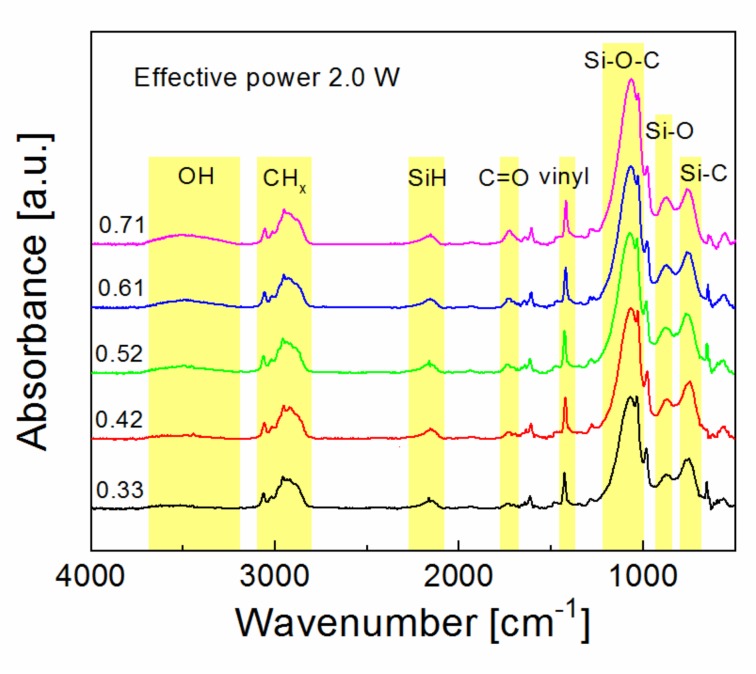
Infrared spectra for plasma nanocoatings deposited at different oxygen fractions from TVS/O_2_ mixture.

Oxidized plasma nanocoatings prepared from the TVS/O2 mixture could therefore be advantageous for nanocoating adhesion. However, the critical load decreased slightly from 1.5 to 1.3 mN with increased oxygen fraction (0–0.71) for plasma nanocoatings deposited at an effective power of 2.0 W (Figure 9). The corresponding FTIR spectra are shown in Figure 10. We can see that all spectra are similar. Oxygen atoms were incorporated into nanocoating during the plasma process and form a Si–O–C network with side-hydroxyl and -carbonyl groups bonded to this network. The concentration of all these species, related to the integral intensity of the absorption band, was partially increased with increased oxygen fraction, which is advantageous for improving the interfacial adhesion at both nanocoating interfaces, since the concentration of the vinyl groups (1590, 1402, 1007, and 951 cm^−1^) was not affected by the oxygen fraction. The expected improved adhesion at the nanocoating/glass interface for oxidized nanocoatings does not correspond to the trend of the critical load in Figure 9.

**Figure 11:**
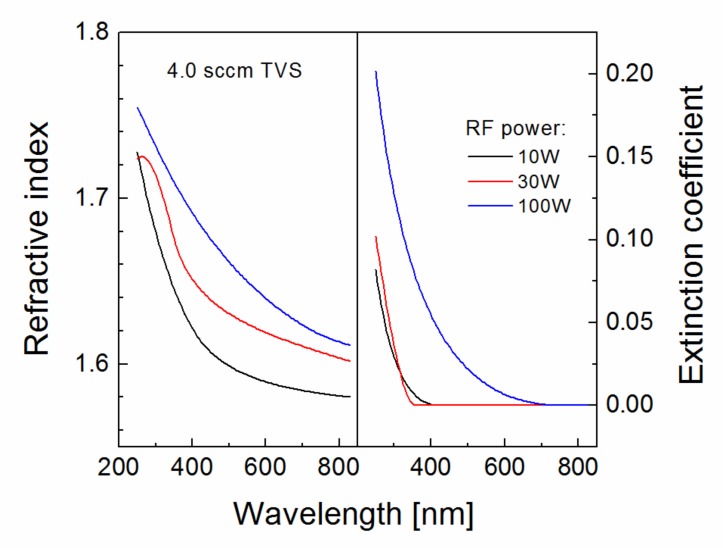
Dispersion curves for the refractive index and the extinction coefficient corresponding to plasma nanocoatings deposited at different RF powers from pure TVS precursor.

Dispersion curves for the refractive index and the extinction coefficient were determined from ellipsometric spectra for plasma nanocoatings deposited on silicon wafers at selected powers (10–100 W) from pure TVS precursor (Figure 11). The refractive index was shifted to higher values at enhanced RF power (left in Figure 11), which corresponds to increased nanocoating density of and also to an increase in mechanical properties (Young’s modulus and hardness) [19]. As the RF power increases, the TVS molecules are fragmented into increasing number of smaller radicals that recombine forming a more crosslinked and denser network resulting in higher mechanical properties of plasma nanocoatings [19]. It was found that the Young’s modulus of plasma nanocoating correlates with its refractive index for a selected wavelength of 633 nm [20] and therefore, the refractive index range (1.59–1.63) in Figure 11 can be assigned to the Young’s modulus range (9.1–14.8 GPa) with enhanced power from 10 to 100 W. These values correspond to the mechanical properties of the polymer-like materials and are consistent with those determined by nanoindentation measurements [21]. Polymer-like nanocoatings synthetized by plasma nanotechnology are characterized by a high yield strength at a sufficiently low Young’s modulus and as such are significant for surface modification of GFs used in GF/polyester composites [17]. The extinction coefficient is simply related to the absorption coefficient and this means that the plasma nanocoatings deposited at lower powers (10 and 30 W) were transparent for visible light (extinction coefficient k = 0), because the absorption edge was below 400 nm (right in Figure 11). Dispersion curves for the refractive index and the extinction coefficient corresponding to the plasma nanocoatings deposited from the TVS/O2 mixture were similar to those for 10 W in Figure 11.

### 3.3. Shear Properties of Glass-Fiber (GF)/Polyester Composites

**Figure 12:**
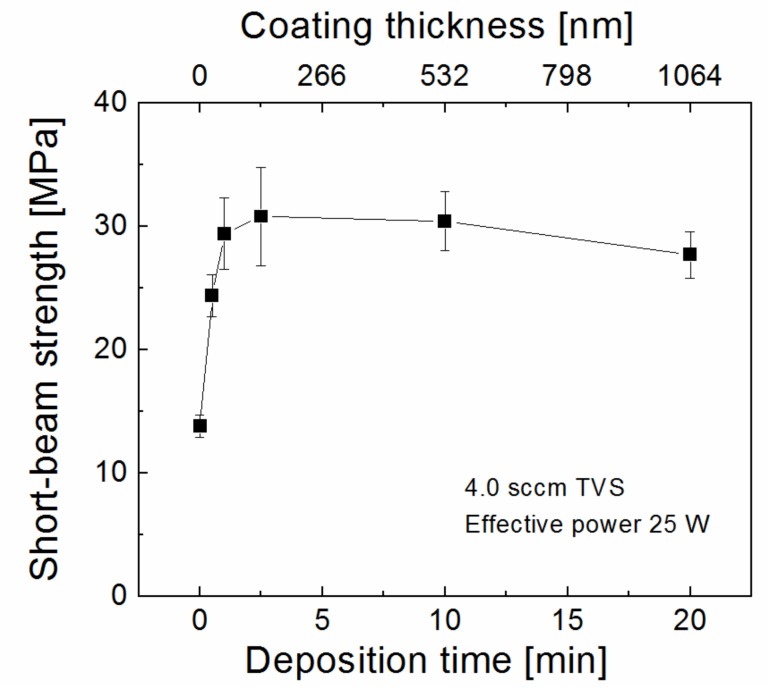
Short-beam strength of glass-fiber (GF)/polyester composites reinforced with GFs, which were plasma-coated at different deposition times and thus with different coating thicknesses.

The bundles of unsized GFs were plasma coated in a tubular PECVD reactor. During the plasma process, the fragments of precursor molecules not only form the plasma nanocoating on the surface of fibers at the edge of the bundle, but also diffuse into the bundle to form the plasma nanocoating on the inner GFs at the center of the bundle. The deposition rate decreases in the direction towards the center of the bundle due to shielding of adjacent fibers. Multiple shielding is controlled by *t*_n_ = *t*_s ._ *q*^n−1^, where *t*_s_ is the film thickness on the fiber at the edge of the bundle, *t*_n_ is the film thickness on the n-th fiber in the direction toward the center of the bundle shielded by n–1 fibers, n = 22 for a bundle with 1600 single filaments, and *q* is the shielding factor of 0.9 [21]. The shielding effect analysis for a bundle with 1600 filaments predicts that the thickness of the plasma nanocoating on the central fiber is only one tenth of the thickness of the nanocoating on the fiber at the edge of the bundle. The GF bundle was plasma coated using a pure TVS precursor at 4.0 sccm and an effective power of 25 W for a different deposition time of 0–20 min. The corresponding coating thickness for the fiber at the edge of the bundle at a deposition rate of 53.2 nm min^−1^ was 0–1064 nm. Untreated and plasma-coated GFs were used to make the GF/polyester composite in a form of short beams. These composite short beams were characterized by the SBS test to evaluate the short-beam strength (Equation (1)), which corresponds to the maximum shear stress of composite failure in interlaminar shear mode [23]. The short-beam strength of GF/polyester composite as a function of deposition time and coating thickness is given in Figure 12. We can see that the short-beam strength increased from 13.8 MPa for untreated GFs to a maximum of 30.8 MPa, which corresponds to a coating of 133 nm (2.5 min) on the surface of fibers at the edge of the bundle, followed by a slight decrease to 27.7 MPa, which is within the error bars and, therefore, not statistically significant. Even 1 min deposition leading to a 53.2 nm-thick coating would appear to be sufficient for the proper functioning of the plasma nanocoating in GF/polyester composite. However, previous analysis of the correct coating thickness was performed using the microindentation test [31], where individual filaments were tested, and the analysis showed that the coating thickness below 100 nm could be insufficient, which would result in lower interfacial shear strength [15,16]. Surface chemical analysis of plasma-coated GFs with a coating thickness below 100 nm revealed that some internal filaments in the bundle were not covered with the continuous coating [16]. Therefore, we decided that a coating thickness of 150 nm on fibers at the edge of the bundle would be sufficient for proper surface modification of GFs and this thickness was used for other plasma coatings.

**Figure 13:**
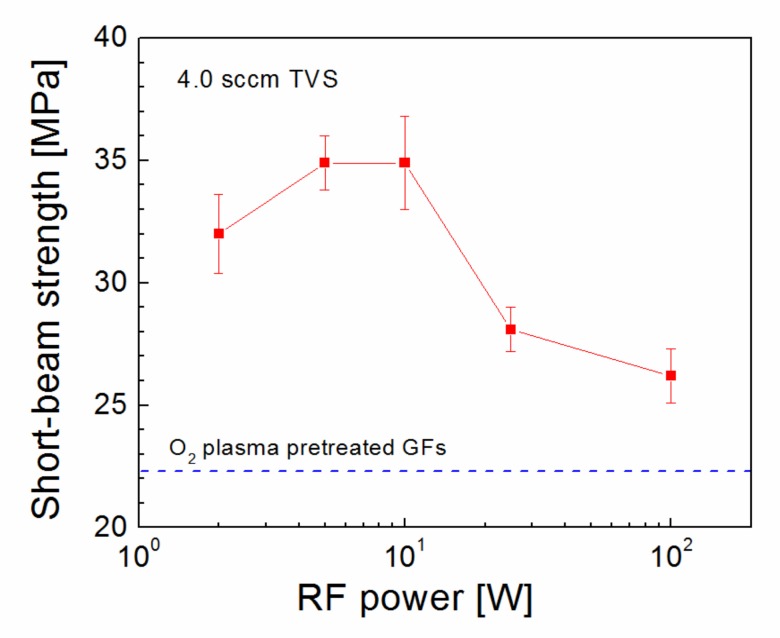
Short-beam strength of GF/polyester composites dependent on RF power used for plasma-coated GFs from pure TVS precursor compared to the shear strength corresponding to oxygen-plasma pretreated GFs.

Plasma nanocoatings deposited from pure TVS precursor at 4.0 sccm and an enhanced RF power of 2.0–100 W were used for surface modification of GFs, which were embedded in polyester resin to produce short composite beams. The short-beam strength of these composites depends on the RF power as shown in Figure 13. First, the strength increased from 32.0 MPa (2.0 W) to a maximum of 34.9 MPa (5 and 10 W), but then significantly decreased to 28.1 and 26.2 MPa for 25 and 100 W, respectively. This decrease is surprising because the interfacial adhesion at the nanocoating/glass interface (Figure 7) does not suggest any such decrease, although an increased Young’s modulus (9.1–14.8 GPa) obtained from the refractive index change is considered (Figure 11). The reason could be a failure at the other interface between the nanocoating and the polyester resin because the concentration of the vinyl groups, responsible for the chemical bond at the polyester/nanocoating interface, decreased significantly with enhanced power (Figure 8). The short-beam strength for plasma-coated GFs was compared with the strength for oxygen-plasma pretreated GFs, where no coating was deposited, and the corresponding value of 22.4 MPa was indicated in Figure 13. Model simulations have clarified that an appropriate interlayer (nanocoating) is required for proper interphase functionality between the fiber reinforcement and the polymer matrix in polymer–matrix composites [16].

**Figure 14:**
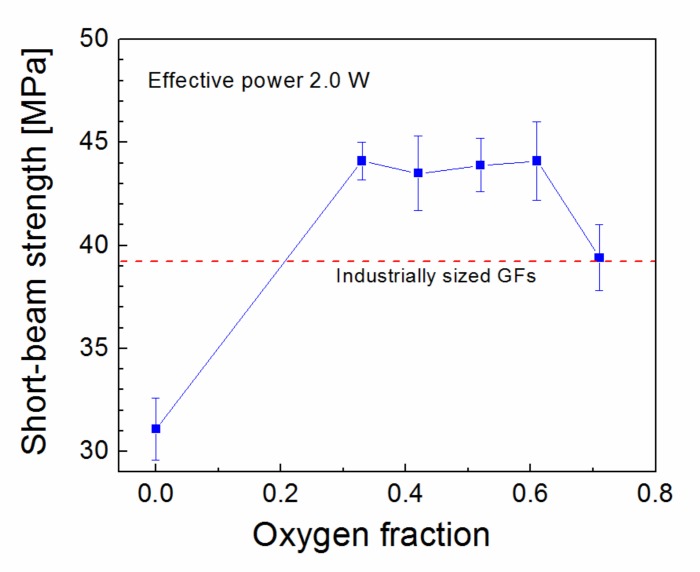
Short-beam strength of GF/polyester composites dependent on oxygen fraction in TVS/O_2_ mixture used for plasma-coated GFs compared to the shear strength corresponding to industrially sized GFs.

**Figure 15:**
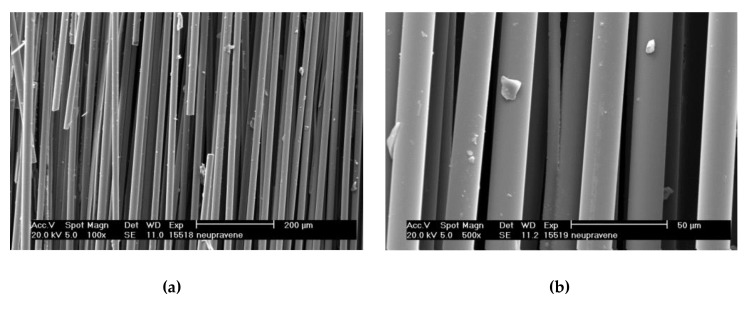
(**a**) Scanning electron microscope (SEM) micrograph of fractured composite beam for weaker interfacial adhesion between the GFs and the polyester resin; (**b**) detailed view.

**Figure 16:**
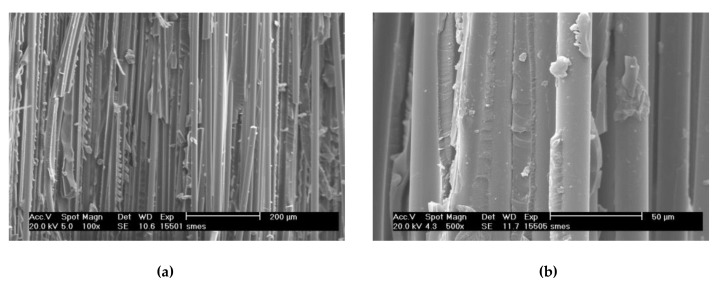
(**a**) SEM micrograph of fractured composite beam for stronger interfacial adhesion between the GFs and the polyester resin; (**b**) detailed view.

Oxidized plasma nanocoatings prepared from the TVS/O2 mixture at an effective power 2.0 W were also deposited on GFs to analyze the effect of oxide species at both nanocoating interfaces on the shear properties of GF/polyester composite. The short-beam strength of these composites was plotted against the oxygen fraction (0–0.71) in TVS/O2 mixture in Figure 14. The shear strength of GF/polyester composites with GFs coated with oxidized plasma nanocoatings was significantly increased compared to GFs coated with non-oxidized nanocoatings (Figure 13). This means that a shear strength of 31.1 MPa (zero oxygen fraction) was increased sharply to a higher level of 43.5–44.1 MPa for a wide range of oxygen fraction of 0.33–0.61 and then decreased to 39.4 MPa for the highest oxygen fraction of 0.71. The steep increase was not predicted by the critical load (Figure 9) for the corresponding oxidized nanocoatings, but could be explained by decreasing the Young’s modulus with enhanced oxygen fraction due to reduced crosslinking of Si–O–C network as found in a recent study [19]. This assumption of increased adhesion at the nanocoating/glass interface is supported by an increased concentration of Si–O–C species as described above (Figure 10). In contrast, the increased concentration of carbonyl groups for the highest oxygen fraction (0.71 in Figure 10) could be responsible for some decrease in shear strength due to reduced interfacial adhesion, where the formation of carbonyl groups prevents covalent bonding to the glass surface as justified in recent studies [16,17,25]. The maximum shear strength for oxidized plasma nanocoatings was 13% higher than 39.2 MPa for industrially sized GFs indicated in Figure 14. Typical load-displacement curves measured during the SBS test for the unsized (untreated), industrially sized, and plasma-coated GFs were previously shown [21]. Short composite beams that failed in interlaminar shear mode were delaminated and the spot, where the failure occurred in the plane of the reinforcement, was observed by SEM. SEM micrographs of fractured composite beams corresponding to unsized (untreated) GFs or poor adhesion at polyester/glass interface (oxygen-plasma pretreated GFs) are given in Figure 15. Figure 15a shows that even with weak interfacial adhesion between the GFs and the polyester resin some fibers were broken, but the fibers were bare and the polyester resin was almost completely removed. Only a few resin residues can be found on the fiber surface as seen in detailed view (Figure 15b). In the case of stronger interfacial adhesion (2.0 W, 0.71 oxygen fraction), the fibers were fully covered with a polyester resin that formed the so-called hackles between the fibers in Figure 16a and in detailed view (Figure 16b). These hackles are typical of interlaminar mode II shear loading.

## 4. Conclusions

Plasma nanotechnology was used to synthetize plasma nanocoatings that could be applied as compatible interlayers in GF/polyester composites with controlled shear properties. For this purpose, plasma processes were investigated in a novel plasma apparatus with a tubular reactor to select suitable deposition conditions for surface modification of GFs in the form of plasma nanocoatings deposited on the glass surface. Based on previous studies, a pure TVS precursor and its mixture with oxygen gas were used as input vapors for atomic processes in non-thermal plasma. Basic plasma characteristics confirmed that all deposition processes took place in a precursor-deficient mode characterized by reduced process pressure. Although the deposition rate along the tubular reactor was shown to be different, the chemical and physical properties of the deposited nanocoatings were approximately consistent and independent of reactor position. The optical properties of plasma nanocoatings indicated a low Young’s modulus (9.1–14.8 GPa) and thus the polymer-like character of the deposited material. Two sets of deposition conditions were selected, which were used for surface modification of GFs applied in GF/polyester composite. The first set corresponded to the use of a pure TVS precursor at an RF power of 2.0–100 W and the second set was concerned to use the TVS/O_2_ mixture at a low effective power of 2.0 W, but a different oxygen fraction of 0–0.71. Plasma-coated GFs were embedded unidirectionally in polyester resin to produce short composite beams. These composite beams were analyzed by the SBS test to determine the short-beam strength, i.e., the shear strength of the composite, which characterizes the functionality of the plasma nanocoatings in GF/polyester composite. A nanocoating thickness of 150 nm was chosen to be sufficient for proper surface modification of all GFs in a 1600 fiber bundle. The short-beam strength varied between 26.2–34.9 MPa depending on RF power and was related to the concentration of the vinyl groups at the polyester/nanocoating interface for plasma nanocoatings deposited from pure TVS precursor. Surface treatment of GFs using oxygen plasma without plasma nanocoatings was not sufficient for efficient stress transfer from the polyester resin to the GFs and the corresponding short-beam strength was only 22.4 MPa. Oxidized plasma nanocoatings deposited from the TVS/O_2_ mixture at 2.0 W allowed changing the short-beam strength from 31.1 to 44.1 MPa depending on the oxygen fraction. This increased shear strength was associated with improved interfacial adhesion between the plasma nanocoating and the glass surface due to the increased concentration of Si–O–C species. Further optimization of deposition conditions with respect to the applied power and the oxygen fraction could extend the range of controllable shear strength.
